# High-Efficiency Multi-Channel Orbital Angular Momentum Multiplexing Enabled by the Angle-Dispersive Metasurface

**DOI:** 10.3390/s24010228

**Published:** 2023-12-30

**Authors:** Ying Li, Qiang Xia, Jun Yang, Guangsheng Deng, Zhiping Yin

**Affiliations:** Special Display and Imaging Technology Innovation Center of Anhui Province, Academy of Opto-Electric Technology, Hefei University of Technology, Hefei 230009, China; liying87@hfut.edu.cn (Y.L.); xq19990428@163.com (Q.X.); junyang@hfut.edu.cn (J.Y.); zpyin@hfut.edu.cn (Z.Y.)

**Keywords:** orbital angular momentum, multiplexing, multi-channel, metasurface, angular dispersion

## Abstract

Orbital angular momentum (OAM) multiplexing of electromagnetic (EM) waves is of great significance for high-speed wireless communication and remote sensing. To achieve high-efficiency OAM multiplexing for multi-channel incident EM waves, this paper presents a novel angle-dispersive meta-atom structure, which can introduce the required anti-symmetric phase dispersion as well as high transmission efficiency for OAM multiplexing. These meta-atoms are then arranged delicately to form an angle-dispersive metasurface working at the X band, which enables three-channel OAM multiplexing by converting highly directional transverse-magnetic (TM) waves incident from 0 and ±45° to coaxial OAM beams with *l* = 0 and ±2 modes, respectively. The simulation and experimental results reveal that the proposed metasurface can convert a higher proportion of energy to the required OAM modes compared to the conventional OAM multiplexing metasurfaces, which can significantly improve the coaxial transmission efficiency of multi-channel OAM multiplexing.

## 1. Introduction

In the development of wireless communication with EM waves, it has always been a key point to provide a higher transmission capacity with the limited channel resources. Since the concept of OAM was first proposed in 1992 [1], a lot of researchers have been inspired to find its potential application in rapid and high-efficiency transmission of EM waves. The EM wave carrying OAM is a phase vertex beam, which can be described by a phase cross section of *e^jlφ^*. Since the topological charge *l* can be any integer, the number of OAM modes is theoretically unlimited, and different OAM modes are initially orthogonal to each other. Due to these fantastic characteristics, multiple EM waves with different frequencies, polarizations, or spatial directions can be converted to OAM beams with orthogonal modes for coaxial transmission [2,3,4,5,6,7,8,9,10,11,12,13,14,15,16,17,18]. This OAM multiplexing method offers a new degree of freedom in addition to the frequency, polarization, phase, and amplitude to manipulate the wireless signals, which can significantly increase the transmission capacity of wireless communication and remote sensing [19,20].

**Figure 1:**
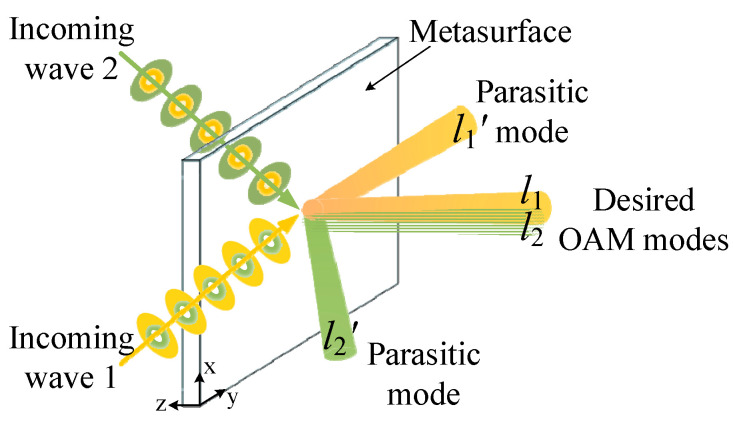
The schematic diagram of a conventional multi-channel OAM multiplexing metasurface.

Metasurfaces, as two-dimensional uniform or diverse metamaterial structures with subwavelength thickness, have been well developed and widely used to manipulate the wavefront, power, or polarization distribution of EM waves [21,22,23,24,25]. A variety of fantastic functionalities have been enabled by metasurfaces, including anomalous reflection/refraction [26], focusing [27], collimation [28], beam steering [29], and OAM generation [30]. In recent years, multi-channel OAM multiplexing for EM waves incident from different directions has also been implemented by metasurfaces [6,7,8,9,10,11,12,13,14,15,16,17,18]. Maguid et al. put forward a shared-aperture metasurface for multi-channel OAM multiplexing [6]. Khonina et al. proposed a field-superposition method to generate multiple Gauss-Laguerre modes with different indices [7]. Based on this method, a lot of field-superposition metasurfaces have been developed for multi-channel OAM multiplexing and demultiplexing [8,9,10,11,12,13,14,15]. Wang et al. used a linear-to-circular polarization converter as metasufaces for multi-channel OAM multiplexing [16]. Zheng et al. adopted OAM multiplexing metasufaces for polarization detecting [17,18]. Although multi-channel OAM multiplexing has been implemented, these metasufaces do not behave the same in the exact phase distribution for OAM multiplexing under each incident direction. The shared-aperture metasurfaces consist of several regions with different phase distributions, and each region exhibits the specific phase distribution to generate a single OAM mode under a single incident direction [6]. On the other hand, the phase distribution of the field-superposition metasurfaces is calculated by summing the field of all required OAM modes under different incident directions [7,8,9,10,11,12,13,14,15]. In this way, these metasurfaces can only convert part of the EM energy from each incoming direction to the desired OAM mode for coaxial transmission, while the rest of the energy is converted to parasitic OAM modes towards other directions, as shown in Figure 1. This phenomenon results in low coaxial transmission efficiency for OAM multiplexing, which limits its application.

In order to overcome this essential problem, an angle-dispersive metasurface is proposed in this work to multiplex multi-channel EM waves incident from axisymmetric directions as orthogonal OAM beams with high coaxial transmission efficiency. To fulfill the exact phase distribution as well as the high-efficiency transmission for OAM multiplexing under each incident direction, a series of Huygens’ meta-atoms is presented, whose angular phase dispersion can be attributed to the asymmetric structure. Based on these meta-atoms, a metasurface example for three-channel OAM multiplexing is constructed, which can convert highly directional TM waves incident from 0 and ±45° to coaxial OAM beams with *l* = 0 and ±2 modes, respectively. Compared to the existing OAM multiplexing metasurfaces, the proposed metasurface can convert a higher proportion of energy to the required OAM modes towards the coaxial direction, which is demonstrated by the simulation and experimental results. Therefore, this angle-dispersive metasurface can significantly improve the coaxial transmission efficiency of multi-channel OAM multiplexing.

## 2. Metasurface Design

### 2.1. Analysis of Phase Response 

**Figure 2:**
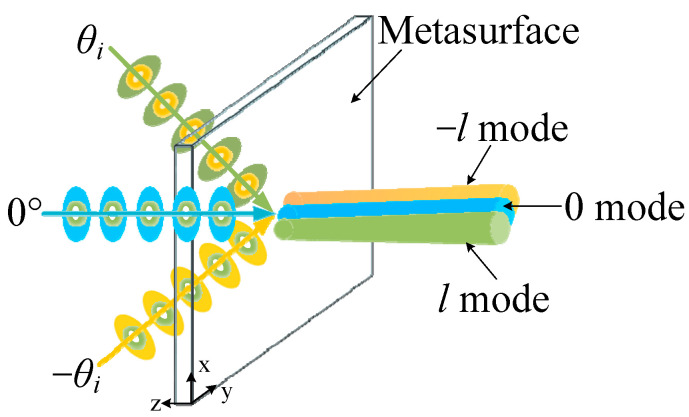
The schematic diagram of a three-channel OAM multiplexing metasurface, which behaves in the exact phase distribution under each incident angle.

To design a metasurface for multi-channel OAM multiplexing, the required phase distribution to generate coaxial OAM beams under each incident direction must be considered first. Here we concentrate on a specific three-channel OAM multiplexing metasurface, which can convert plane waves incident from 0° and ±*θ_i_* to coaxial OAM beams with 0 and ±*l* (*l* can be any integer) modes, respectively, as shown in Figure 2. This metasurface is placed in free space. High-directional beams incident from symmetric angles ±*θ_i_* are converted to OAM beams with opposite modes of ±*l* towards the normal direction (*θ_t_* = 0°), while another beam incident from 0° is also directed to the normal direction with the *l* = 0 mode. Generally, the required phase response Φ of this metasurface consists of two parts:

Φ = Φ_⊥_ + Φ_OAM_, (1)

where Φ_⊥_ is the required phase for normal transmission and Φ_OAM_ is the required phase for OAM generation. According to the generalized Snell’s law of refraction [31] and the theory of OAM generation [32], the required Φ_⊥_ and Φ_OAM_ under ±*θ_i_* incidence can be calculated as,

Φ_⊥_ (±*θ_i_*) = ±2π*x_i_* · sin*θ_i_*, (2)

Φ_OAM_ (±*θ_i_*) = ±*l* · tan^−1^ (*y_i_*/*x_i_*) + Φ_0_. (3)

Here, *x_i_* and *y_i_* denote the position of the *i*th meta-atom in Cartesian coordinates, and Φ_0_ denotes a constant phase. So, in order to multiplex two beams from symmetric incident angles (±*θ_i_*) with two opposite OAM modes (±*l*), the transmission phase response of each meta-atom should be anti-symmetrically angle-dispersive as,

Φ(*θ_i_*) − Φ_0_ = Φ_0_ − Φ(−*θ_i_*). (4)

In particular, each meta-atom should behave in the constant phase of Φ = Φ_0_ to generate the *l* = 0 mode under normal incidence. 

### 2.2. Design of Meta-Atoms

**Figure 3:**
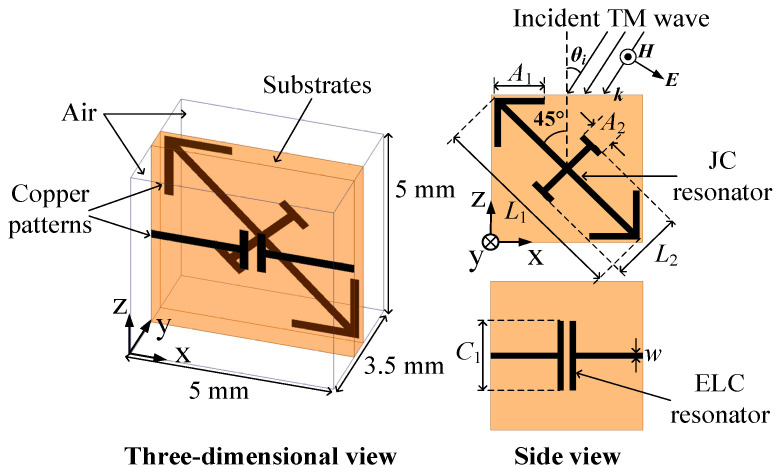
The detailed structure of the proposed meta-atoms.

In our previous work, the asymmetric Huygens’ meta-atom structure has been utilized to achieve anti-symmetric angular phase dispersion as well as high transmission efficiency, which is composed of multi-layer substrates patterned with electric-field-coupled (ELC) resonators, titled split-ring resonators (SRRs), or Jerusalem-cross (JC) resonators [33,34]. However, the multi-layer structure is weighty, and the bonding films between adjacent layers of substrates are unavoidable, which bring about a more complicated design and assembling procedure. Also, the multi-layer structure means a higher dielectric and metallic loss, resulting in low transmission efficiency. To overcome these problems, a simplified asymmetric Huygens’ meta-atom structure is proposed in Figure 3 to achieve the required phase and amplitude distribution for TM-wave OAM multiplexing. This meta-atom structure is implemented by a single layer of substrate perpendicular to the transverse magnetic field, which is easy to fabricate and assemble. The substrate is patterned by an ELC resonator and an arrow-typed JC resonator on the opposite sides. The JC resonator exhibits unequal cross strips *L*_1_ and *L*_2_ and a tilted angle of 45° from the normal incident direction to ensure the asymmetric structure. The longer strip *L*_1_ is connected with an arrow at the end to extend its equivalent length, which can significantly enlarge the angular phase dispersion under symmetric incident angles. 

Based on the surface equivalence principle, the transmission and reflection coefficients of a Huygens’ metasurface composed of the given meta-atom are [35],

*T* = (4 − *Y*_es_*Z*_ms_)/[(*Y*_es_*η* + 2)(*Z*_ms_/*η* + 2)], *R* = 2(*Z*_ms_/*η* − *Y*_es_*η*)/[(*Y*_es_*η* + 2)(*Z*_ms_/*η* + 2)], (5)

where *Y*_es_*η* and *Z*_ms_/*η* are the normalized electric admittance and magnetic impedance, respectively. By carefully adjusting the strip lengths *L*_1_, *L*_2_, and *C*_1_, as well as the arrow lengths *A*_1_ and *A*_2_, an equal relation of *Y*_es_*η* = *Z*_ms_/*η* can be simultaneously achieved at the resonant frequency under the normal incidence of TM waves. Given the lossless substrate material and metallic patterns, then high transmission efficiency can be achieved within a frequency band around the resonance. When the incident angle is gradually shifted from 0° to ±*θ_i_*, the JC and ELC resonators exhibit changed equivalent lengths. So, the resonant frequency band will also shift with a change in the incident angle, and the overlap of these resonant frequency bands results in a shared resonant frequency band for high-efficiency transmission across the incident angle range. Meanwhile, due to the asymmetric structure and the high angular phase response correlation of the meta-atom, the disparate values of *Y*_es_*η* and *Z*_ms_/*η* under different incident angles will bring about the required anti-symmetric angular phase dispersion within the same band.

**Figure 4:**
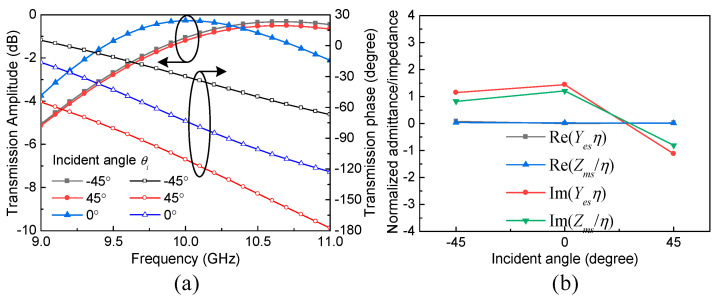
(**a**) The transmission amplitude and phase performance of the meta-atom sample; (**b**) the values of *Y*_es_*η* and *Z*_ms_/*η* at 10 GHz within −45° ≤ *θ_i_* ≤ 45°.

To verify the above analysis, an optimized meta-atom sample working at around 10 GHz is presented, whose dimension is 5 × 3.5 × 5 mm^3^. The chosen substrate is Rogers RT/duroid 6010LM (*ε*_r_ = 10.2, tan*δ* = 0.0023) with a thickness of 0.635 mm, which is patterned by copper resonators on both sides with a thickness of 0.017 mm. The geometric parameters of the JC and ELC resonators are *L*_1_= 6.5 mm, *L*_2_ = 0.5 mm, *C*_1_ = 2.86 mm, *A*_1_ = 0.55 mm, and *A*_2_ = 0.2 mm, while the width of each copper strip is *w* = 0.2 mm. Full-wave simulation by the commercial electromagnetics solver, Ansoft HFSS, has been adopted to find the transmission amplitude and phase performance of this meta-atom, which is illustrated in Figure 4a. It can be seen that high transmission and anti-symmetric angular phase dispersion can be simultaneously achieved within a shared resonant frequency band of 9.8–10.5 GHz across the incident angle range of −45° ≤ *θ_i_* ≤ 45°. The values of *Y*_es_*η* and *Z*_ms_/*η* at 10 GHz are retrieved according to Equation (5) in Figure 4b, which are almost pure imaginary and approximately within −45° ≤ *θ_i_* ≤ 45°, resulting in the high transmission efficiency. Meanwhile, the values of *Y*_es_*η* and *Z*_ms_/*η* are changed within −45° ≤ *θ_i_* ≤ 45°, which lead to the anti-symmetric angular phase dispersion. These transmission properties can fulfill the requirement of OAM multiplexing for TM waves.

**Figure 5:**
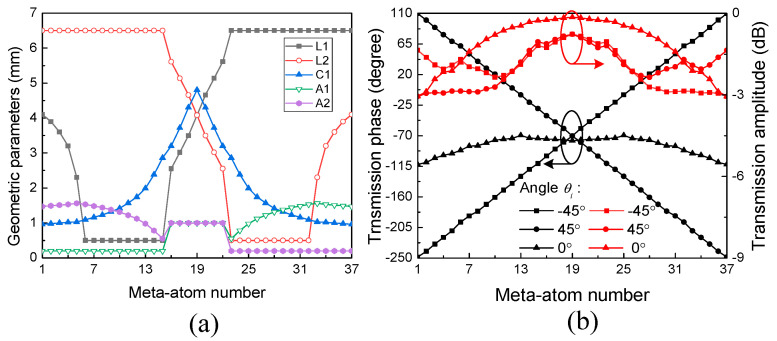
(**a**) The proper values of geometric parameters for a series of 37 meta-atoms; (**b**) the simulated transmission phase and amplitude of each meta-atom under *θ_i_* = 0° and ±45°.

Based on this meta-atom structure, a series of 37 meta-atoms with different geometric parameters are designed to achieve different ranges of angular phase dispersion at 10 GHz. It has been demonstrated in our previous work that by increasing the lengths of *L*_1_ and *A*_1_, and simultaneously decreasing the lengths of *L*_2_ and *C*_1_, the meta-atom structure behaves in a wider range of angular phase dispersion [34]. Conversely, a smaller range of angular phase dispersion can be achieved by the opposite operation of these parameters. The optimized values of *L*_1_, *L*_2_, *C*_1_, *A*_1_, and *A*_2_ for these meta-atoms are presented in Figure 5a, while the transmission amplitude and phase of each meta-atom under *θ_i_* = 0° and ±45° are displayed in Figure 5b. The central meta-atom (number 19) is selected to be the reference meta-atom, with symmetric geometric parameters *L*_1_ = *L*_2_ and *A*_1_ = *A*_2_ to guarantee the almost constant phase Φ_0_, while the parameters’ values of the other meta-atoms are asymmetrically distributed about the central meta-atom to obtain opposite ranges of angular phase dispersion under *θ_i_* = ±45°. It can be found that the insertion loss of most meta-atoms is less than 3 dB, and their transmission phase response can cover 360° with anti-symmetric angular phase dispersion about Φ_0_ = −70° under *θ_i_* = ±45°. Meanwhile, the transmission phase under *θ_i_* = 0° behaves with a small deviation from the constant of Φ_0_. Therefore, these meta-atoms are appropriate to constitute an angle-dispersive metasurface for three-channel OAM multiplexing under *θ_i_* = 0° and ±45°.

In addition, if the oblique incident angle *θ_i_* is less than ±45°, these 37 meta-atoms will exhibit smaller ranges of angular phase dispersion, thus they cannot cover the 360° phase response anymore. In this situation, for each meta-atom, the values of *L*_1_ and *A*_1_ should be increased, and the values of *L*_2_ and *C*_1_ should be simultaneously decreased to enlarge the angular phase dispersion range. In this way, the optimized geometric parameters of these 37 meta-atoms can be regenerated to cover the 360° phase response.

### 2.3. Implemention of the Functional Metasurface

**Figure 6:**
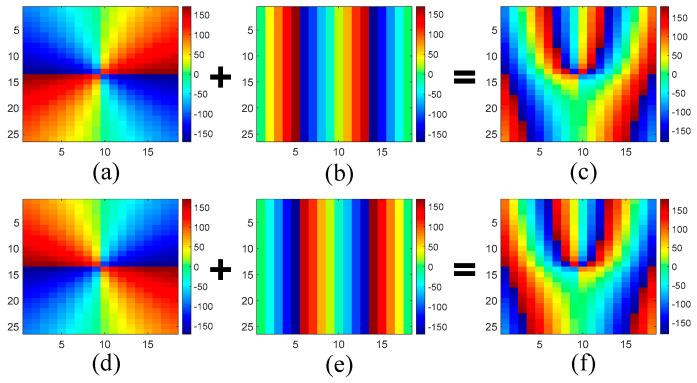
(**a**) The required phase response Φ_OAM_ for OAM generation under *θ_i_* = −45°; (**b**) the required Φ_⊥_ for normal transmission under *θ_i_* = −45°; (**c**) the desired total phase distribution under *θ_i_* = −45°; (**d**) the required Φ_OAM_ for OAM generation under *θ_i_* = 45°; (**e**) the required Φ_⊥_ for normal transmission under *θ_i_* = 45°; (**f**) the desired total phase distribution under *θ_i_* = 45°.

**Figure 7:**
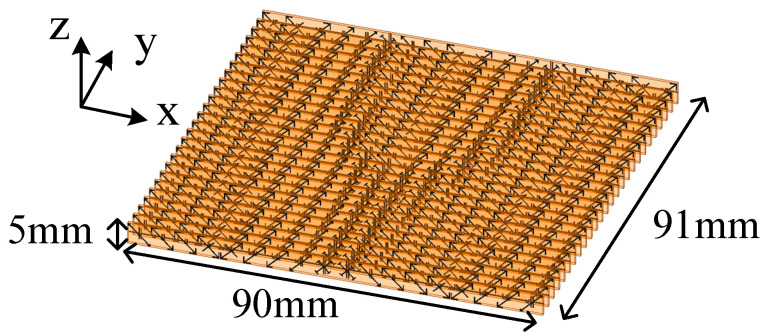
The whole structure of the three-channel OAM multiplexing metasurface.

**Figure 8:**
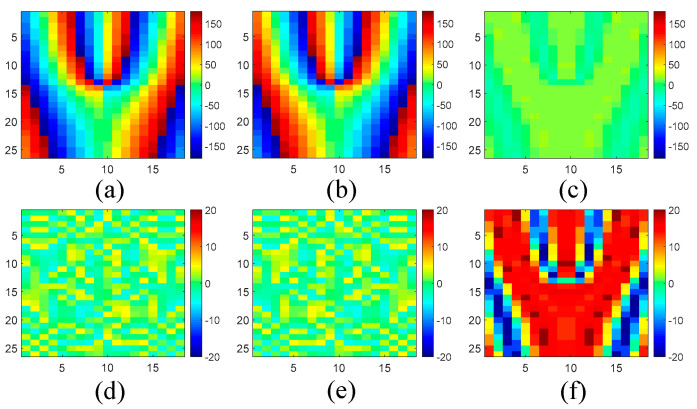
(**a**) The realized phase distribution under *θ_i_* = −45°; (**b**) the realized phase distribution under *θ_i_* = 45°; (**c**) the realized phase distribution under *θ_i_* = 0°; (**d**) the phase error under *θ_i_* = −45°; (**e**) the phase error under *θ_i_* = 45°; (**f**) the phase error under *θ_i_* = 0°.

Based on the above meta-atoms, a functional metasurface for three-channel OAM multiplexing can be constructed. As a proof of concept, a metasurface prototype is presented to multiplex three beams incident from 0° and ±45° with orthogonal OAM modes of *l* = 0 and ±2 to the normal direction, respectively. This metasurface is composed of 18 × 26 different meta-atoms with a total dimension of 90 × 91 × 5 mm^3^. For the meta-atoms at different positions of the metasurface, the required phase response Φ_⊥_ for normal transmission and Φ_OAM_ for OAM generation under *θ_i_* = ±45° are calculated according to Equations (2) and (3). Then, both of them are summed together to obtain the desired total phase distribution, as shown in Figure 6. In addition, the desired phase distribution under normal incidence should be a constant of Φ_0_ to achieve the *l* = 0 mode. According to the desired phase distribution, the meta-atoms which behave in the right phase response under each incident angle are carefully arranged to the proper positions. As a result, the whole structure of the metasurface is constructed in Figure 7. The realized phase distribution of the metasurface and the phase error compared to the ideal values are shown in Figure 8. It can be found that the maximum phase error under *θ_i_* = 0° is about ±20°, while the phase error under *θ_i_* = ±45° is no more than ±5°. Meanwhile, the average phase errors under these incident angles are also calculated by,

(6) $$\bar{\Phi_{error}}=\frac{1}{mn}\sum_{i=1}^{m}\sum_{j=1}^{n}\left|\Phi_{ij\_real}-\Phi_{ij\_ideal}\right|,$$

where *m* and *n* are the total number of meta-atoms in the *x* and *y* direction, and Φ*_ij__*_real_ and Φ*_ij__*_ideal_ are the realized and ideal phase response of the *i*th and *j*th meta-atom in the *x* and *y* direction, respectively. The calculated average phase errors are 12.4° under *θ_i_* = 0° and 2.5° under *θ_i_* = ±45°. So, the proposed metasurface exhibits similar phase distribution as the ideal values under each incident direction. 

## 3. Results and Discussion

**Figure 9:**
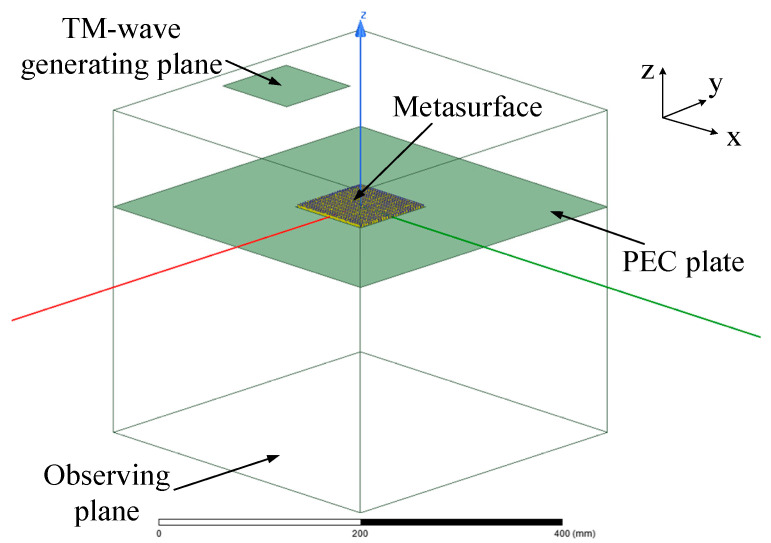
The simulation model of the presented metasurface for multi-channel OAM multiplexing.

**Figure 10:**
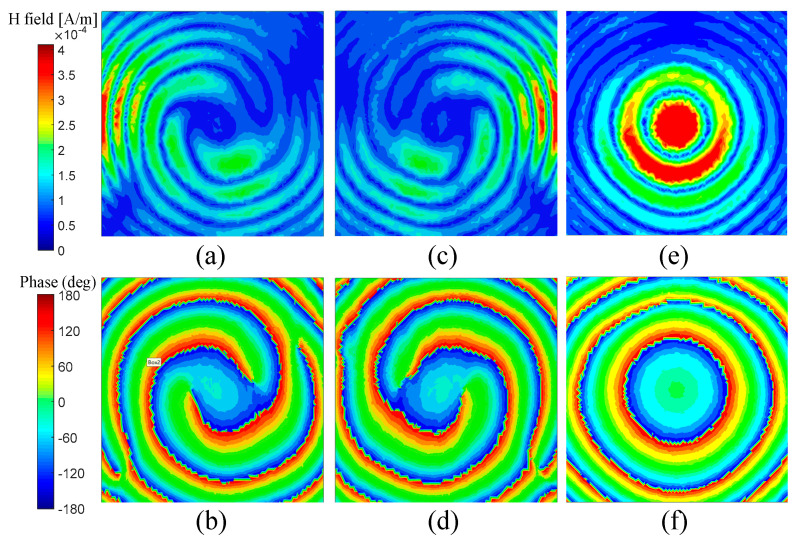
The simulated amplitude and phase distribution at 10 GHz on the observing plane: (**a**) the amplitude and (**b**) phase distribution under *θ_i_* = −45°; (**c**) the amplitude and (**d**) phase distribution under *θ_i_* = 45°; (**e**) the amplitude and (**f**) phase distribution under *θ_i_* = 0°.

To demonstrate the OAM multiplexing performance of the presented metasurface, full-wave simulation by Ansoft HFSS has been conducted. As illustrated in Figure 9, the metasurface model is placed in the *x-o-y* plane and surrounded by a plate, which is set to be the perfect electric conductor to eliminate the interference by the diffraction wave. A TM-polarized plane wave, whose transverse magnetic field is perpendicular to the *x-o-z* plane, is generated to illuminate the metasurface from three symmetric incident angles: 0° and ±45°. A parallel square plane right under the center of the metasurface with a separation of 300 mm is used to observe the distribution of the transmitted TM wave. The simulated phase and amplitude distribution at 10 GHz on the observing plane are displayed in Figure 10. It can be seen that the phase distribution under *θ_i_* = ±45° exhibits two centrosymmetric vortexes, which are clockwise or counterclockwise, respectively. The corresponding amplitude is distributed as four centrosymmetric vortexes. So, the phase and amplitude distribution under ±45° coincide with that of the *l* = ±2 OAM modes, respectively. Meanwhile, the phase and amplitude distribution under *θ_i_* = 0° looks like concentric circles, which agrees with that of the *l* = 0 OAM mode. These simulation results indicate that the metasurface model has the function of three-channel OAM multiplexing.

**Figure 11:**
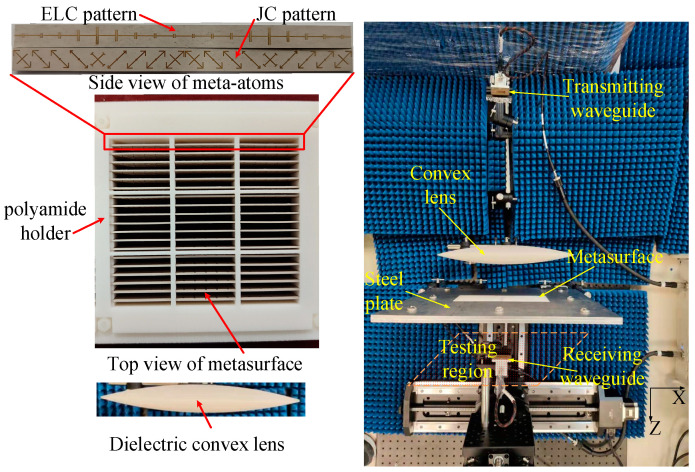
The configuration of the metasurface prototype and its experimental setup.

Then, a prototype of the proposed metasurface is fabricated and tested to experimentally verify the OAM multiplexing performance. This prototype consists of 18 × 26 meta-atoms, which are fabricated by etching the proper copper patterns on the Rogers RT/duroid 6010LM laminates. These laminates are fixed on a 3D-printed polyamide holder to ensure each meta-atom is firm enough and placed in the accurate position. Figure 11 shows the configuration of the metasurface prototype and its experimental setup. A WR-90 waveguide is used to radiate spherical TM waves at the X band. A dielectric convex lens made of Teflon is adopted to concentrate the spherical TM wave as a highly directional TM wave. The metasurface prototype with the holder is embedded in the center of a steel plate to eliminate the interference by the diffraction wave. Then, the waveguide and convex lens are rotated around the center of the metasurface in the *x-o-z* plane to achieve different illumination angles. The transmitted TM wave is tested by another WR-90 waveguide moving in the testing region, whose distance to the metasurface is set to be 300 mm. 

**Figure 12:**
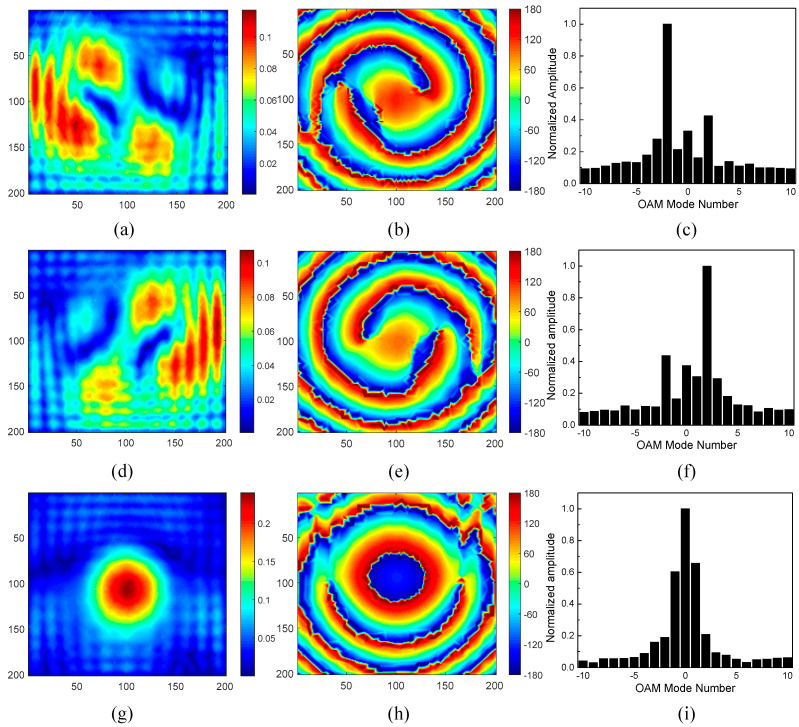
The measured E-field distribution and mode spectra at 10 GHz in the testing region: (**a**) the amplitude and (**b**) phase distribution under *θ_i_* = −45°; (**c**) mode spectra under *θ_i_* = −45°; (**d**) the amplitude and (**e**) phase distribution under *θ_i_* = 45°; (**f**) mode spectra under *θ_i_* = 45°; (**g**) the amplitude and (**h**) phase distribution under *θ_i_* = 0°; (**i**) mode spectra under *θ_i_* = 0°.

The measured E-field distribution at 10 GHz in the testing region under the illumination angles of 0° and ±45° is plotted in Figure 12. The measured amplitude is uneven under *θ_i_* = ±45°, which is due to the unbalanced transmission amplitude of meta-atoms placed in different locations and the diffracted power through the gap between the metasurface and the steel plate. The measured phase distribution exhibits similar centrosymmetric vertexes under *θ_i_* = ±45° and quasi-concentric circles under *θ_i_* = 0° compared to the simulated results, which nearly coincides with that of the OAM modes *l* = ±2 and *l* = 0, respectively. The differences between the simulated and measured phase distribution can also be attributed to the leakage of incident power directly through the gap between the metasurface and the steel plate. In addition, the mode spectra of the proposed metasurface under each incident angle are calculated based on the measured data and are also shown in Figure 12. It can be found that the dominant OAM modes under *θ_i_* = −45°, 0°, and 45° are *l* = −2, 0, and 2, respectively. In this way, the three-channel OAM multiplexing capability of the proposed metasurface is experimentally verified.

Furthermore, to evaluate the coaxial transmission efficiency of the proposed metasurface for OAM multiplexing, the power proportion multiplexed to the desired OAM mode towards the normal direction should be taken into account. Since our proposed metasurface behaves in the exact phase distribution for OAM multiplexing under each incident angle, its multiplexed power proportion (denoted by *P*) is equal to the mode purity (denoted by *P_m_*), which can be calculated from the measured mode spectra [36],

*P* = *P_m_* = *A_l_*^2^/∑ *A_i_*^2^. (7)

Here, *A_l_* is the normalized amplitude of the desired OAM mode, and *A_i_* is the normalized amplitude of the *i*th OAM mode. From the measured mode spectra shown in Figure 10, it can be calculated that the mode purities for OAM modes *l* = −2, 0, and 2 are 21%, 24%, and 22%, respectively, which indicate that the multiplexed power proportion of the proposed metasurface is larger than 21%.

On the other hand, because the realized phase distribution of a conventional *n*-channel OAM multiplexing metasurface is generally calculated by the superposed field of all required OAM modes, a number of *n* OAM beams towards different directions with almost equal power will be simultaneously generated by this metasurface under each incident angle, and only one beam exhibits the desired OAM mode towards the normal direction, as illustrated in Figure 1. So, the multiplexed power proportion of a conventional *n*-channel OAM multiplexing metasurface can be approximately calculated by,

*P* = *P_m_*/*n*, (8)

which is much smaller than its mode purity. 

Table 1 shows the comparison of the multiplexed power proportion of our proposed metasurface and conventional OAM multiplexing metasurfaces [8,9,10,11,12,13,14,15,16]. It can be found that the lowest multiplexed power proportion of our proposed metasurface (*P* = 21% @ *l* = −2) is still much higher than that of most conventional OAM multiplexing metasurfaces. From this comparison, it can be concluded that our proposed metasurface is appropriate for high-efficiency multi-channel OAM multiplexing.

## 4. Conclusions

In summary, a kind of angle-dispersive metasurface is presented in this work to multiplex multi-channel beams incident from axisymmetric directions as orthogonal OAM beams with high coaxial transmission efficiency. This metasurface is composed of single-layer asymmetric Huygens’ meta-atoms, which can introduce the required anti-symmetric phase dispersion as well as high transmission efficiency for OAM multiplexing. As a proof of concept, a metasurface prototype is designed and fabricated, which can convert three-channel TM waves incident from 0 and ±45° to coaxial OAM beams with *l* = 0 and ±2 modes, respectively. The three-channel OAM multiplexing performance of this prototype is confirmed by simulation and experimental results. Compared to the conventional OAM multiplexing metasurfaces, the proposed metasurface has the exact required phase distribution under each incident angle, thus it exhibits a higher proportion of multiplexing power to the desired OAM modes towards the normal direction. As a result, this angle-dispersive metasurface can significantly improve the coaxial transmission efficiency of three-channel OAM multiplexing. With this design method, we can construct similar metasurfaces for three-channel OAM multiplexing with symmetric incident angles 0 and ±*θ_i_* (0° < *θ_i_* < 45°) and symmetric topological charges 0 and ±*l* (*l* can be any integer). Some practical applications of this kind of OAM multiplexing metasurface include high-capacity wireless communication, high-resolution polarization detection, and remote sensing.

## Figures and Tables

**Table 1:** Comparison of the multiplexed power proportion of our proposed metasurface with other reports.

Ref.	Phase Distribution Strategy	Multiplexed OAM Modes	Mode Purity (*P_m_*)	Multiplexed Power Proportion (*P*)
[8]	Field summation	0, ±1, ±2	Not provided	Not provided
[9]	Field summation	±1, ±2	Not provided	Not provided
[10]	Field summation	−1, −2	Not provided	Not provided
[11]	Field summation	±4, ±5	≤53%	≤13.3%
[12]	Field summation	1, 2	≤28.6%	≤14.3%
[15]	Field summation	2, 4	≤62%	≤31%
[16]	Polarization division	±1	Not provided	Not provided
This work	Exact calculation	0, ±2	≥21%	≥21%

## Data Availability

Data are contained within the article.
